# Detecting the existence of gene flow between Spanish and North African goats through a coalescent approach

**DOI:** 10.1038/srep38935

**Published:** 2016-12-14

**Authors:** Amparo Martínez, Arianna Manunza, Juan Vicente Delgado, Vincenzo Landi, Ayotunde Adebambo, Muritala Ismaila, Juan Capote, Mabrouk El Ouni, Ahmed Elbeltagy, Asmaa M. Abushady, Salah Galal, Ainhoa Ferrando, Mariano Gómez, Agueda Pons, Bouabid Badaoui, Jordi Jordana, Oriol Vidal, Marcel Amills

**Affiliations:** 1 Departamento de Genética, Universidad de Córdoba, Córdoba 14071, Spain; 2 Department of Animal Genetics, Center for Research in Agricultural Genomics (CSIC-IRTA-UAB-UB), Campus Universitat Autònoma de Barcelona, Bellaterra 08193, Spain; 3 Department of Animal Breeding and Genetics, Federal University of Agriculture, Abeokuta PMB 2240, Nigeria; 4 Instituto Canario de Investigaciones Agrarias, La Laguna 38108, Tenerife, Spain; 5 Livestock & Wildlife Laboratory, Arid Land Institute Medenine, 4119 Médenine, Tunisia; 6 Department of Animal Biotechnology, Animal Production Research Institute, Dokki, Giza, Egypt; 7 Genetics Department, Faculty of Agriculture, Ain Shams University, Shubra 11241, Cairo, Egypt; 8 Animal Production Department, Faculty of Agriculture, Ain Shams University, Abbassia 11566, Cairo, Egypt; 9 Departament de Ciència Animal i dels Aliments, Universitat Autònoma de Barcelona, Bellaterra 08193, Spain; 10 Servicio de Ganadería. Diputación Foral de Bizkaia. Avda. Lehendakari Aguirre n° 9-2°, 48014 Bilbao, Spain; 11 Unitat de Races Autòctones, Servei de Millora Agrària, (SEMILLA-SAU), Son Ferriol 07198, Spain; 12 University Mohammed V, Agdal, Faculty of Sciences, 4 Av. Ibn Battota, Rabat, Morocco; 13 Departament de Biologia, Universitat de Girona, Girona 17071, Spain

## Abstract

Human-driven migrations are one of the main processes shaping the genetic diversity and population structure of domestic species. However, their magnitude and direction have been rarely analysed in a statistical framework. We aimed to estimate the impact of migration on the population structure of Spanish and African goats. To achieve this goal, we analysed a dataset of 1,472 individuals typed with 23 microsatellites. Population structure of African and Spanish goats was moderate (mean F_ST_ = 0.07), with the exception of the Canarian and South African breeds that displayed a significant differentiation when compared to goats from North Africa and Nigeria. Measurement of gene flow with Migrate-n and IMa coalescent genealogy samplers supported the existence of a bidirectional gene flow between African and Spanish goats. Moreover, IMa estimates of the effective number of migrants were remarkably lower than those calculated with Migrate-n and classical approaches. Such discrepancies suggest that recent divergence, rather than extensive gene flow, is the main cause of the weak population structure observed in caprine breeds.

During three millenia, cattle, goats and sheep originally domesticated in the Fertile Crescent, followed human Neolithic migrations, probably reaching the Iberian Peninsula and the Maghreb by 7,700 YBP and 7,000 YBP^1^, respectively. Cyprus is thought to have been colonized by Northern Levant seafarers, who brought the four major livestock species (cattle, sheep, goats and pigs), approximately 9,000–10,500 YBP^1^. In Mediterranean Europe, rather than a gradual transition from the Mesolithic to the Neolithic lifestyles, evidence suggests a sharp demographic decrease of Late Mesolitic cultures and the settlement of Neolithic colonists at previously unhabited coastal locations^1^. The current view is that this migratory movement did not follow a constant pace *i.e*. it took 2,000 years to move from Cyprus to the Aegean, another 500 years to establish in Italy, and 500–600 additional years to reach the Iberian Peninsula^1^.

North Africa was also a fundamental corridor for the diffusion of animal species domesticated in the Fertile Crescent^2^,^3^,^4^. Zooarchaeological evidence suggests that cattle, sheep and goats were raised at the Sahara desert during the African Humid Period^2^. Circa 5,900 YBP, a dramatic climatic shift triggered a process of desertification that forced African agropastoral societies to move southwards^2^,^3^. Nomad populations of Central Saharan herdsmen arrived to the Sahel probably around 4,000 YBP^2^. Subsequently, nomad pastoralists may have entered South Africa through the Tsetse-free corridor down the highland spine of East Central Africa^2^. This migratory wave likely took place 2,000–2,400 YBP^2^.

The analysis of mitochondrial and Y-chromosome^4^,^5^ markers suggests that the African and Iberian caprine genetic pools remained connected by gene flow after their post-domestication split, but this has not been formally demonstrated in a statistical framework yet. In the current work, we have examined the magnitude and direction of migration between goat populations from Africa and Spain by analysing, with classical methods and coalescent genealogy samplers, a dataset of ~34,000 microsatellite genotypes. Our goal was to assess, in a statistical modeling framework, if migration had a relevant role in shaping the patterns of caprine genetic diversity found in Africa and the Iberian Peninsula.

## Results

### Analysis of genetic diversity and population structure

Most of the continental breeds analysed in the current work displayed high levels of diversity, with H_e_ values above 0.60 (Supplementary Table S1). With regard to insular populations, H_e_ estimates were 0.68, 0.63–0.68 and 0.49–0.66 in Cape Verdean, Balearic and Canarian goats, respectively. We also observed a tendency towards an increased diversity in Northwest African and Egyptian goats when compared with the Spanish ones *i.e*. the three populations with highest H_e_ (>0.70) were the Baladi, Saidi and Tunisian ones. In contrast, H_e_ estimates were particularly low in the Palmera (H_e_ = 0.49) and Tinerfeña del Norte (H_e_ = 0.57) breeds.

In general, the majority of F_ST_ coefficients (Supplementary Table S2) were low (F_ST_ < 0.05) or moderate (F_ST_ = 0.05–0.15), providing evidence that population structure is moderate (mean F_ST_ = 0.07) but significant (*P* < 0.001). There were, however, a few cases in which F_ST_ values reflected marked genetic differentiation. Indeed, when we compared the South African and Canarian goats with the remaining populations, estimated F_ST_ coefficients were moderate (F_ST_ = 0.06–0.14) but highly significant (*P* < 1.10^−5^). The comparison of goats from Spain and Central West Europe (Alpine and Saanen are French and Swiss breeds, respectively) and Northwest Africa (Supplementary Table S2), revealed a low level of differentiation (F_ST_ ≈ 0.03–0.04 for both comparisons), and the same result was obtained when we compared goat populations from Northwest Africa and Nigeria (F_ST_ ≈ 0.03). Insular goat breeds showed various patterns of differentiation with mainland populations. For example, Canarian goats appeared equally differentiated from African and Spanish breeds. In contrast, Balearic goats displayed a moderate differentiation with their Spanish (F_ST_ = 0.01–0.03) and Northwest African (F_ST_ ≈ 0.05) counterparts, respectively (Supplementary Table S2).

**Figure 1:**
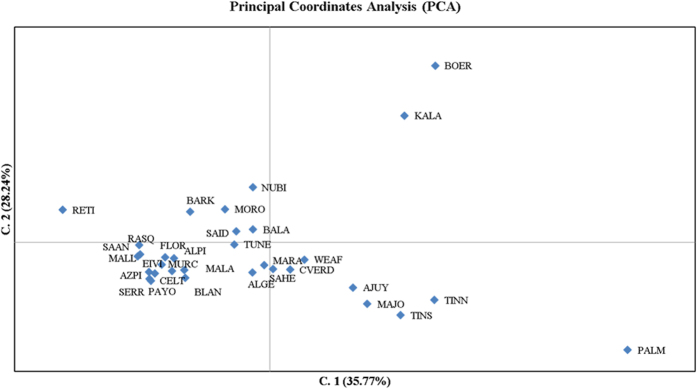
Principal coordinates analysis of 32 goat populations based on a pairwise F_ST_ matrix. Populations under analysis are: **Southern Spain**: Blanca Andaluza (BLAN), Blanca Celtibérica (CELT), Malagueña (MALA), Murciano-Granadina (MURC), Florida (FLOR), Payoya (PAYO), Negra Serrana (SERR), Retinta (RETI); **Northern Spain**: Azpi-Gorri (AZPI), Blanca de Rasquera (RASQ); **Balearic Islands**: Eivissenca (EIVI), Mallorquina (MALL); **Canary Islands**: Ajuy (AJUY), Majorera (MAJO), Palmera (PALM), Tinerfeña del Norte (TINN), Tinerfeña del Sur (TINS); **Cape Verde** (CVER); **Central West Europe**: Saanen (SAAN), Alpine (ALPI); **Northwest Africa**: Moroccan (MORO), TUNE (Tunisian), Algerian (ALGE); **Egypt**: Barki (BARK), Baladi (BALA), Saidi (SAID); **Nigeria**: Maradi (MARA), West African Dwarf (WEAF), Sahel (SAHE); **South Africa**: Boer (BOER), Kalahari Red (KALA). The Anglo-Nubian breed (mixed British, African and Indian origins) is indicated as NUBI.

The Principal Coordinates Analysis (PCoA) plot shown in Fig. 1 supported the high differentiation of South African and Palmera goats when compared with the remaining caprine populations. Peninsular Spanish, Balearic and Central West European (Alpine and Saanen) breeds grouped together forming a small cluster clearly distinguishable from the African one. Northwest African and Egyptian breeds were distributed in a scattered cluster that was placed close to the Nigerian and Cape Verdean populations. A third highly differentiated cluster was formed by the Canarian breeds.

**Figure 2:**
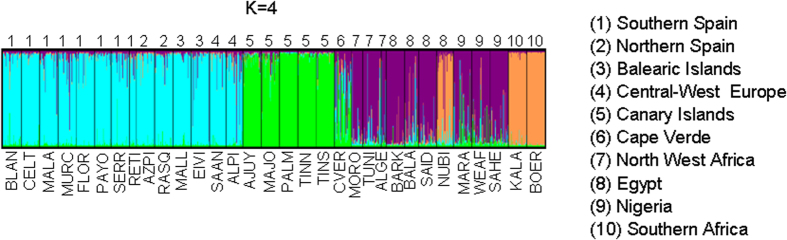
Structure analysis of 32 goat populations on the basis of the most significant K-value (K = 4), as inferred with the method reported by Evanno^34^. The complete analysis (K = 2–6) can be found in Supplementary Fig. S1. **Southern Spain**: Blanca Andaluza (BLAN), Blanca Celtibérica (CELT), Malagueña (MALA), Murciano-Granadina (MURC), Florida (FLOR), Payoya (PAYO), Negra Serrana (SERR), Retinta (RETI); **Northern Spain**: Azpi-Gorri (AZPI), Blanca de Rasquera (RASQ); **Balearic Islands**: Eivissenca (EIVI), Mallorquina (MALL); **Canary Islands**: Ajuy (AJUY), Majorera (MAJO), Palmera (PALM), Tinerfeña del Norte (TINN), Tinerfeña del Sur (TINS); **Cape Verde** (CVER); **Central West Europe**: Saanen (SAAN), Alpine (ALPI); **Northwest Africa**: Moroccan (MORO), TUNE (Tunisian), Algerian (ALGE); **Egypt**: Barki (BARK), Baladi (BALA), Saidi (SAID); **Nigeria**: Maradi (MARA), West African Dwarf (WEAF), Sahel (SAHE); **South Africa**: Boer (BOER), Kalahari Red (KALA). The Anglo-Nubian breed (mixed British, African and Indian origins) is indicated as NUBI.

Bayesian clustering of the populations with Structure (Fig. 2, Supplementary Fig. S1) was consistent with the main trends outlined above. At all explored K-values (three independent runs were made for each K-value), the Canarian breeds formed a distinctive and homogeneous cluster clearly differentiated from the remaining ones. At K = 3 (Supplementary Fig. S1) and at K = 4 (Fig. 2), we detected a split between Spanish, Balearic and Central West European goats and their African counterparts. Importantly, Spanish breeds displayed the same genetic background as Alpine and Saanen goats even at K = 5 (Supplementary Fig. S1). Moreover, the majority of Spanish breeds were poorly differentiated. The African cluster became evident at K = 3 (Supplementary Fig. S1) and at K = 5 it was further subdivided into (1) South African breeds (Boer and Kalahari Red), (2) Egyptian (Barki, Baladi, Saidi) and Anglo-Nubian goats, and (3) Northwest African and Nigerian populations. The Structure analysis (K = 3–4) also revealed signals of mixed Spanish, Canarian and African genetic backgrounds in the Cape Verdean goats. This Atlantic archipelago remained uninhabited until Portuguese sailors discovered it in the 15^th^ century. Afterwards, Portuguese, West African and Canarian livestock were imported into Cape Verde as a source of food, a circumstance that may explain our findings.

We also computed the coefficients of membership at the most significant K-value *i.e*. K = 4 (Supplementary Fig. S3). We found that Spanish and Central West European breeds shared the same genetic background. Moreover, the genetic background more prevalent in Northwest African, Egyptian and Nigerian breeds (Supplementary Fig. S3) was weakly present (~4–6%) in Spanish goats (North Spain, South Spain and Balearic Islands), though it is difficult to assess the significance of this finding because this faint signal might not be due to a post-domestication introgression event but to ancient shared ancestry amongst goat populations. In Africa, there was a clear split between Northwest African and Nigerian goats with regard to those from the Southern part of the continent, although Egyptian breeds shared ancestry with the latter (at K = 4, 35%). The Canarian breeds had a unique genetic background, completely different from that found in other breeds, whilst, as previously discussed, goats from Cape Verde had a mixed Canarian, Spanish and African ancestry.

### Estimation of migration rates

We estimated migration rates with the softwares Migrate-n^6^ and IMa^7^. With Migrate-n (Supplementary Table S3), the average Spearman correlation between parameter estimates obtained in two independent runs was 0.98, thus indicating that we had reached convergence. We did not know the mutation rates of the microsatellites employed in our analysis, so we did not make any attempt to convert our parameter estimates into quantities expressed in demographic units. Indeed, the main objectives of our study could be fully achieved without making such conversions. For the four migration routes investigated here, migration rate 95% confidence intervals did not comprise the zero-value.

With regard to IMa parameter estimates (Supplementary Tables S4 and S5 and Supplementary Fig. S4), the effective sample sizes and autocorrelation values obtained plus the comparison between two independent M-mode runs (Supplementary Fig. S4) demonstrated that our analyses had reached convergence. In general, θ-estimates obtained with IMa were larger than those inferred with Migrate-n, and this was particularly true for the Egyptian and Northwest African population. In contrast, migration rates inferred with IMa were much lower and more asymmetric than those calculated with Migrate-n (Supplementary Tables S3 and S4). We also calculated mutation-scaled times of divergence between populations as well as the sizes of the ancestral populations that yielded them (Supplementary Table S5).

The comparison of IMa nested models by using likelihood ratio tests (threshold of significance after correction for multiple testing, *P*-value = 0.003) showed that, in all cases, the full migration model (bi-directional asymmetric migration) had a much better fit to the data than the reduced model without migration (indicated by significant P-values in Table 1). In contrast, in several cases the full model did not account for the data significantly better than simpler models. For instance, when we analysed data from Northwest and Egyptian goats, the full model did not account for the data better than models with symmetric bidirectional migration or unidirectional migration (Table 1). The calculation of N_e_m with four methods, namely Wright’s equation^8^, the private-alleles method^9^ and Migrate-n^6^ and IMa^7^ coalescent genealogy samplers, showed remarkable discrepancies (Table 2). By far, the F_ST_ method yielded the largest N_e_m estimates, while the method of private-alleles and Migrate-n showed intermediate N_e_m values and IMa the smallest ones.

## Discussion

We detected a high level of variation in the majority of African and Spanish goat breeds, with H_e_ in the range of 0.60–0.70 (Supplementary Table S1). These values were consistent with previous estimates obtained in South East Asian^10^ (H_e_ = 0.30–0.71), European and Near Eastern^11^ (H_e_ = 0.69), Indian (H_e_ = 0.73–0.78)^12^ and Chinese^13^ (H_e_ = 0.61–0.78) goat breeds. We also observed a limited level of genetic differentiation between Northwest African (Morocco, Algeria, Tunisia), Egyptian and Nigerian goat populations (F_ST_ ≈ 0.03–0.06). In the PCoA plot (Fig. 1), they grouped in relatively close proximity and in the Structure analysis (Fig. 2 and Supplementary Fig. S1) they displayed a similar genetic background. These results may seem paradoxical because North Africa and Nigeria are separated by the Sahara desert, a formidable geographic barrier to human and livestock dispersal. However, the Imazighen people that inhabit the Sahara are pastoral nomads that have traversed the desert during millenia transporting goods and livestock^2^. Moreover, in the Early Holocene (9,000–5,900 YBP) the Sahara was not the hyper-arid desert of present times, but a savanna ecosystem with a benign climate that supported herding activities^2^.

The population structure of African goats was mostly explained by the strong genetic differentiation between South African breeds (Boer and Kalahari Red) and those from Northwest Africa and Nigeria (Figs 1 and 2, Supplementary Fig. S1). Marked genetic differences between goats from South Africa and Mozambique have been observed when comparing them with those from North and West Africa^4^. Similarly, clear differentiation has been demonstrated between Southern African Pafuri and Ndebele breeds with regard to those from West and East Africa^14^. It would be worth investigating if the Tsetse fly belt (latitude parallels 15°N to 29°S) has enhanced the genetic differentiation of South African breeds by limiting genetic exchanges with northern areas. In this regard, an analysis of the landscape genetics of Burkina Faso goats provided evidence that the most significant genetic discontinuity between goat populations coincided with the boundary between Tsetse fly infested and free areas^15^. Indeed, trypanosomiasis could have affected the patterns of genetic diversity of African goats not only by acting as a biological barrier to the diffusion of trypanosusceptible goats but also because of the long-term selection pressure for trypanotolerance on goats raised in infested areas.

Data presented in Table 2 provided compelling evidence that F_ST_ coefficients give, in all cases, much higher N_e_m estimates than those provided by coalescent genealogy samplers. There are various possible explanations for this discrepancy. When the assumptions of the Wright approximation^8^, *i.e*. infinite number of populations at migration-drift equilibrium, mutation rates much lower than migration rates and absence of selection, are not met, N_e_m tends to be overestimated. Moreover, this lack of correspondence between F_ST_ and N_e_m is exacerbated when F_ST_ values are small^16^. Although F_ST_ should be considered as an excellent measure of genetic differentiation, it is clear that it does not tell much about the relative weight of its causal factors.

We used two coalescent genealogy samplers to calculate migration rates and N_e_m (Table 2, Supplementary Tables S3 and S4). The comparison of both parameter estimates made evident that migration rates obtained with IMa are lower than those inferred with Migrate-n. Similar observations were made in previous studies^17^,^18^, where IMa estimates of migration were, at some instances, two orders of magnitude lower than those obtained with Migrate-n. This outcome is probably due to the fact that these two coalescent methods are based on different assumptions and statistical models^6^,^7^. Indeed, divergence times of goat breeds are intrinsically short (<10,000 YBP) because they descend from a single gene pool domesticated in Eastern Anatolia, a feature that is modeled by IMa but not by Migrate-n.

Our assessment is that IMa gives more accurate measurements of migration rates because it takes into account the existence of isolation (*i.e*. the sharing of alleles is not only due to migration but also to recent divergence). Using this software, we compared full and reduced models (Table 1). For all routes, models with zero migration in both directions had a much poorer fit to the data than the full models. In other words, our results provided evidence that migration is statistically significant for the four migratory routes under analysis.

Regarding the accuracy of our migration estimates, we should acknowledge that our experimental design violates some assumptions of the IMa method, such as the lack of population substructure and the potential existence of unsampled populations. Although IMa has been shown to be quite robust to small to moderate violations of the Isolation with Migration Model, gene flow with unsampled populations could bias upward the splitting time between populations and the effective size of the ancestral population and also increase the asymmetry in the direction of gene flow^19^. Despite the fact that our experimental design does not perfectly match all the assumptions of IMa and this could lead to some bias in parameter estimates, we do not expect this circumstance to substantially modify the main conclusions of our study.

Detecting gene flow between Europe and Africa has been a major subject of research in the fields of human and livestock population genetics^20^,^21^,^22^. The results obtained here suggest the existence of migration between Southern Spain and Northwest Africa (Tables 1 and 2 and Supplementary Tables S3 and S4). This result is consistent with previous data showing the existence of genetic connections between the Maghreb and Iberian caprine gene pools^4^. Moreover, the analysis of ancient bovine remains at the archaeological site of Atapuerca (northern Spain) demonstrated the presence of a mitochondrial variant with a likely African origin^21^. We also found significant gene flow from Central West Europe to Northern Spain, a finding that agrees well with their recent common ancestry^3^.

The impact of African introgression into Spanish breeds seemed to be quite limited. When we estimated the proportion of Central West European (Alpine and Saanen) *vs* Northwest African genetic background in the genomes of peninsular Spanish goats by using Structure, we only found weak traces of a putative African ancestry (4–6%, Supplementary Fig. S3). It is difficult to judge the significance of this finding, though it is worth highlighting that a recent analysis of worldwide bovine diversity indicated that the magnitude of African introgression into Iberian cattle was around 7.5%^23^. This limited admixture is consistent with the significant genetic differentiation that exists between Southern Spanish and North African both goats^4^ and cattle^23^. We can conclude that after dispersal from the Eastern Anatolia domestication center, the caprine Spanish and Northwest African gene pools evolved mostly in an independent manner, though some genetic exchanges took place.

We anticipated the existence of gene flow between the Canary Islands and Northwest Africa because this archipelago was settled by Imazighen peoples around 3,000 YBP, as supported by several lines of archaeological, linguistic and genetic evidence^24^, and current Canarian goat populations are thought to descend from the ones brought by the first settlers of the archipelago^25^. We also found significant bidirectional gene flow between Egypt and Northwest Africa (Table 1). In this regard, it is worth highlighting that North Africa has been inhabited over millenia by nomadic pastoralists, such as the Tuaregs and Bedouins, whose economy was mainly based on herding. This involved the seasonal migration of herders and livestock from winter to summer pastures and vice versa (transhumance).

## Conclusions

We have detected the existence of gene flow between North African and Spanish goats, suggesting that these two gene pools did not evolve in a completely independent manner after their split ~7,000 YBP^3^. From a broader perspective, our data show that analysing gene flow in a domestic species as goats, where population splitting times are intrinsically short, without taking into account the contribution of isolation may lead to inflated estimates of migration rates.

## Methods

### Ethics statement

Blood and hair root samples were collected from goats by trained veterinarians in the context of sanitation campaigns and parentage controls not directly related with our research project. For this reason, permission from the Universitat Autònoma de Barcelona Committee of Ethics in Animal Experimentation was not required. In all instances, veterinarians followed standard procedures and relevant national guidelines to ensure appropriate animal care.

### Goat sampling and genotyping

We have used a dataset of 1,472 individuals from 32 goat populations (Supplementary Table S6, Supplementary Fig. S5) native from Northern Spain (N = 106), Southern Spain (N = 303), Balearic Islands (N = 137), Central West Europe (N = 73), Northwest Africa (N = 96), Egypt (N = 156), Nigeria (N = 161), South Africa (N = 93), Canary Islands (N = 310) and Cape Verde (N = 37). Part of this dataset (856 Spanish Peninsular, Balearic and Canarian goats typed with 20 microsatellites) has been recently employed to make inferences about the population structure of Iberian caprine breeds^26^. Genomic DNA purification from blood and hair samples and microsatellite genotyping were performed as previously described^26^. The primers employed in microsatellite amplification are reported in Supplementary Table S7. After inspecting microsatellite data visually as well as with Lositan^27^, Genepop^28^, and MicroChecker^29^, that detect selection, linkage disequilibrium and presence of null alleles respectively, six markers were discarded (*CSSM66, INRA5, ILSTS19, SRCRSP5, SRCRSP23* and *SRCRSP24*) and the remaining 23 were used in further analyses.

### Genetic diversity analyses

Genetic differentiation among breeds was estimated with Arlequin v3.3^30^ by calculating the total and pairwise F_ST_ coefficients. The program GenAlEx v6.5^31^ was utilized to infer expected heterozygosities (H_e_) as well as to generate a PCoA plot based on a pairwise F_ST_ matrix. In order to investigate the population structure of goat breeds, we employed Structure v2.3.4^32^. We carried out 25 runs with 1 million iterations and 200,000 iterations as burn-in. We considered the allele frequencies as correlated and we used the admixture option, considering a range of K-values from 2 to 6. This analysis was carried out three times in order to assess the repeatability of results. The output of Structure was collated with the web-based program Structure Harvester^33^ in order to visualize likelihood scores and to infer the most likely K-value^34^. Coefficients of membership were obtained by averaging the inferred values of ancestry of individuals for each population.

### Inferring gene flow with coalescent genealogy samplers

We used two coalescent genealogy samplers i.e Migrate-n^6^ and IMa^7^ to estimate mutation-scaled population sizes (θ_i_ = 4N_e_μ) and migration rates (M_ij_ = m_ij_/μ). We did not use TreeMix to infer migration events because this software models migration between populations as occurring at a single instantaneous time point^35^, an assumption that is quite unrealistic for domestic species. Genealogy samplers estimate parameters by using a large collection of genealogies^36^. They often employ a Markov Chain Monte Carlo (MCMC) approach to explore the parameter space with the goal of identifying those genealogies that best fit the data^36^. Our coalescent analysis was focused on four potential migratory routes: Egypt vs Northwest Africa, Northwest Africa vs the Canary Islands, Northwest Africa vs Southern Spain and Northern Spain vs Central West Europe. We did not analyse the route linking Northwest Africa with Cape Verde because this archipelago was colonized only 500 YBP, and this time of divergence is too short to allow meaningful migration analyses. We also excluded South African and Nigerian breeds from our analysis because the central and meridional parts of the African continent are poorly sampled in our study.

Migrate-n considers an Island Model where populations have been exchanging migrants at a constant rate for a very long time (island model). In contrast, IMa is built on an Isolation with Migration Model that includes six parameters: three population sizes (q_1_, population 1; q_2_, population 2, and q_A_, ancestral population from which populations 1 and 2 are derived), two migration rates and the splitting time^7^. The Isolation with Migration model implemented in IMa can distinguish between migration and the sharing of ancestral polymorphisms because it explicitly models both factors in a coalescent model where gene trees can coalesce in the ancestral population (common history) and exchange migrants amongst populations (migration) within the same genealogy and with separate parameters that will determine the shape of such genealogy. The more probable genealogy produced by both processes and given the observed data will be accepted. In consequence, significant differences between IMa and Migrate-n estimates (with IMa < Migrate-n) would imply that recent divergence has contributed significantly to allele sharing amongst populations.

#### Migrate-n analysis

Migrate-n v. 3.6. builds on a n-island model where population sizes and migration rates do not change over time, and considers asymmetrical migration rates and populations with distinct sizes^6^. After several optimization pilot runs, we decided to infer migration rates on a pairwise basis (two populations at a time) because if not (*i.e*. when considering all populations at the same time) the computational burden was unmanageable with the available resources. We used subsamples of genotypes retrieved from 30 individuals (per population) and 10 microsatellites chosen at random (*ETH10, ETH225, MAF65, CRSM60, BM6526, TGLA122, SPS115, OarFCB11, OarFCB304* and *McM527*) because otherwise the amount of time required to complete the analyses was prohibitive. We initially made two short independent runs, using a Brownian motion mutation model that approximates the classical stepwise model, and a maximum-likelihood inference strategy. We considered variable mutation rates amongst loci, and the M_ij_ and θ_i_ starting parameters were based on F_ST_ and N_e_ calculations, respectively. The parameter M_ij_ defines the proportion of genes of population j that come from population i per generation. Each of the replicate runs consisted of 10 short-chains (100,000 visited genealogies, 1,000 recorded steps) and three long-chains (4 million visited genealogies, 40,000 recorded steps), with a burn-in of one million iterations. We used an adaptive heating scheme with 4 chains with temperatures set by default at Migrate-n (swapping interval  = 1) to increase the efficiency of the MCMC search. Averaged parameter estimates were used as priors in two subsequent long runs based on an adaptive heating scheme and comprising 10 short-chains (100,000 visited genealogies, 1,000 recorded steps), and three long-chains (8 million visited genealogies, 80,000 recorded steps) with a burn-in of 1 million steps. We made sure that both long runs converged to the same parameter estimates, that were subsequently averaged.

#### Isolation with Migration analysis (IMa)

IMa is better suited than Migrate-n for analysing populations (populations 1 and 2) that diverged recently from a source population (population A) *i.e* less than 10,000 years ago. The migration rate m_1_ defines the fraction of genes of population 1 that come from population 2 (m_1_ would be equivalent to M_21_ of Migrate-n) per generation, while m_2_ refers to the opposite direction (m_2_ = M_12_ of Migrate-n). We carried out pre-analyses of the data to define suitable prior ranges and running conditions. Parameter ranges for uniform priors were chosen on the basis of the highest posterior density intervals (at 90%) of the estimates obtained in these initial runs. Priors for θ-values were used directly as the upper bounds of the corresponding prior distributions and they varied depending on the run under consideration (Supplementary Fig. S4). To ensure an efficient exploration of the parameter space, we employed a geometric heating scheme (heating parameters: g_1_ = 0.9, g_2_ = 0.8) with 200 Metropolis-coupled Markov chains. We made sure that the mixing was good and that convergence had been achieved by checking estimated sample sizes, autocorrelations, and parameter trendline plots (Supplementary Fig. S4). Once we verified that marginal posterior distributions of the two runs had achieved similar solutions, a total of 150,000 genealogies were analysed by using the nested models option in the “Load Trees Mode” (L-Mode). By using log-likelihood ratio tests, this analysis determined if the fully parameterized IMa model explains the data significantly better than a series of simpler models with fewer parameters.

### Calculation of the effective number of migrants (N_e_m)

The effective number of migrants (N_e_m, immigration expressed in units of genetically effective individuals) was calculated by using a variety of methods. The first of them was based on F_ST_ estimates and derives N_e_m with equation (1)^8^:


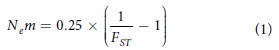


In addition, we calculated N_e_m with the private-alleles method^9^, implemented in Genepop^28^, that takes into account a correction for population size. Equation (2) assumes that there is a linear relationship between the average frequency of private alleles (*p*_*i*_) and N_e_m. Indeed, private alleles (those present in only one subpopulation) may reach high frequencies only when N_e_m is small. The corresponding equation can be expressed as:


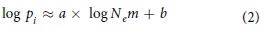


where *a* and *b* are the slope and the intercept of the regression of *N*_*e*_*m* over *p*_*i*_ (both depend on the number of individuals sampled by subpopulation), respectively^37^. Finally, we also inferred N_e_m from coalescent-based estimates (θ_1_, θ_2_, M_21_, M_12_) obtained with Migrate-n and IMa. In this context, N_e_m depends on the product of the effective size of the population that receives the migrants by the corresponding migration rate *i.e*. the fraction of the recipient population composed by immigrants. The relationship between θ_i_ and M_ij_ can be expressed with equation (3) when considering autosomal markers^38^:


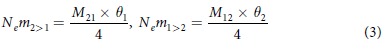


## Additional Information

**How to cite this article**: Martínez, A. *et al*. Detecting the existence of gene flow between Spanish and North African goats through a coalescent approach. *Sci. Rep.* **6**, 38935; doi: 10.1038/srep38935 (2016).

**Publisher's note:** Springer Nature remains neutral with regard to jurisdictional claims in published maps and institutional affiliations.

## Supplementary Material

Supplementary Information

## Figures and Tables

**Table 1:** Statistical support for the full *vs* reduced models comparison inferred with IMa through likelihood ratio tests.

Population 1	Population 2	M_21_ = M_12_		M_21_ ≠ 0, M_12_ = 0		M_2>1_ = 0, M_12_ ≠ 0		M_21_ = M_12_ = 0	
		2LLR	P-value	2LLR	P-value	2LLR	P-value	2LLR	P-value
NSPAIN	CW EUROPE	4.18	0.040	6.76	0.009	7.90	0.004	912.34	**<0**.**001**
SSPAIN	NW AFRICA	11.14	**<0**.**001**	72.24	**<0**.**001**	576.95	**<0**.**001**	909.19	**<0**.**001**
NW AFRICA	EGYPT	4.93	0.026	1.02	0.31	0.20	0.65	242.62	**<0**.**001**
CANARY I.	NW AFRICA	11.00	**<0**.**001**	13.06	**<0**.**001**	354.90	**<0**.**001**	911.48	**<0**.**001**

All models assume that θ_1_, θ_2_ and θ_A_ can have different magnitudes. Degrees of freedom (d.f.) = 1 for all comparisons except the one involving the full migration model *vs* the model without migration (d.f. = 2). M_21_, migration rate from population 2 to population 1; M_12_, migration rate from population 1 to population 2. By using the Bonferroni correction, we have set the level of significance to 0.05/16 = 0.003 (4 migratory routes × 4 model comparisons = 16). Results that are significant after correction for multiple testing are shown in bold. Abbreviations: NSPAIN: Northern Spanish goats, CW EUROPE: Central-Western European goats, SSPAIN: Southern Spanish goats, NW AFRICA: Northwestern African goats EGYPT: Egyptian goats, CANARY I: Canarian goats. 2LLR: twice the log-likelihood ratio.

**Table 2:** Estimates of the effective number of migrants (N_e_m) calculated with Wright equation^8^, Slatkin method^9^, Migrate-n^6^ and IMa^7^.

Population 1	Population 2	F_ST_^a^	F_ST_^b^	Slatkin^a^	Slatkin^b^	Migrate-n^b^		IMa^b^	
		N_e_m	N_e_m	N_e_m	N_e_m	N_e_m_2>1_	N_e_m_1>2_	N_e_m_2>1_	N_e_m_1>2_
NSPAIN	CW EUROPE	5.37	5.56	3.04	3.14	2.21	3.17	0.88	0.00
SSPAIN	NW AFRICA	6.33	7.12	5.43	2.87	3.66	3.40	1.21	0.07
NW AFRICA	EGYPT	6.69	8.78	2.62	1.59	4.61	4.13	0.02	3.53
CANARY I.	NW AFRICA	2.98	2.85	2.05	1.92	1.57	1.07	0.93	0.11
**AVERAGES**		**5**.**34**	**6**.**08**	**3**.**29**	**2**.**38**	**3**.**01**	**2**.**94**	**0**.**76**	**0**.**93**

^a^N_e_m calculated with the whole population data and a microsatellite panel of 23 markers. ^b^N_e_m calculated with a set of 10 microsatellites and sample sizes = 30. Abbreviations: NSPAIN: Northern Spanish goats, CW EUROPE: Central-Western European goats, SSPAIN: Southern Spanish goats, NW AFRICA: Northwestern African goats EGYPT: Egyptian goats, CANARY I: Canarian goats.
