# Supplementary material for: Detecting the existence of gene flow between Spanish and North African goats through a coalescent approach
Source: Sci Rep. 2016 Dec 14;6:38935. doi: 10.1038/srep38935 (PMC5155231; doi:10.1038/srep38935)

**Detecting the existence of gene flow between Spanish and North African goats through a coalescent approach**

^1^Amparo Martínez, ^2^Arianna Manunza, ^1^Juan Vicente Delgado, ^1^Vincenzo Landi, ^3^Ayotunde Adebambo, ^3^Muritala Ismaila, ^4^Juan Capote, ^5^Mabrouk El Ouni, ^6^Ahmed Elbeltagy, ^7^Asmaa M. Abu Shady, ^8^Salah Galal, ^9^Ainhoa Ferrando, ^10^Mariano Gómez, ^11^Agueda Pons, ^12^Bouabid Badaoui, ^9^Jordi Jordana, ^13^Oriol Vidal, ^2^Marcel Amills

^1^Departamento de Genética, Universidad de Córdoba, Córdoba 14071, Spain; ^2^Department of Animal Genetics, Center for Research in Agricultural Genomics (CSIC-IRTA-UAB-UB), Campus Universitat Autònoma de Barcelona, Bellaterra 08193, Spain; ^3^Department of Animal Breeding and Genetics, Federal University of Agriculture, Abeokuta PMB 2240, Nigeria; ^4^Instituto Canario de Investigaciones Agrarias, La Laguna 38108, Tenerife, Spain; ^5^Livestock & Wildlife Laboratory, Arid Land Institute Medenine, 4119 Médenine, Tunisia; ^6^Department of Animal Biotechnology, Animal Production Research Institute, Dokki, Giza, Egypt; ^7^Genetics Department, Faculty of Agriculture, Ain Shams University, Shubra 11241, Cairo, Egypt; ^8^Animal Production Department, Faculty of Agriculture, Ain Shams University, Abbassia 11566, Cairo, Egypt; ^9^Departament de Ciència Animal i dels Aliments, Universitat Autònoma de Barcelona, Bellaterra 08193, Spain; ^10^Servicio de Ganadería. Diputación Foral de Bizkaia. Avda. Lehendakari Aguirre nº 9-2º, 48014 Bilbao, Spain; ^11^Unitat de Races Autòctones, Servei de Millora Agrària, (SEMILLA-SAU), Son Ferriol 07198, Spain; ^12^University Mohammed V, Agdal, Faculty of Sciences, 4 Av. Ibn Battota, Rabat, Morocco; ^13^Departament de Biologia, Universitat de Girona, Girona 17071, Spain;

Supplementary Table S1. Mean observed (H_o_) and expected (H_e_) heterozygosities (with their standard errors) of goats sampled in the current study.

| **Pop** | **H_o_** | **H_e_** |
| --- | --- | --- |
| **Central Werst Europe** | | |
| Saanen | 0.646 ± 0.053 | 0.650 ± 0.046 |
| Alpine | 0.650 ± 0.040 | 0.681 ± 0.037 |
| **Southern Spain** | | |
| Blanca Andaluza | 0.654 ± 0.041 | 0.668 ± 0.037 |
| Blanca Celtibérica | 0.648 ± 0.042 | 0.673 ± 0.039 |
| Malagueña | 0.625 ± 0.038 | 0.677 ± 0.039 |
| Murciano-Granadina | 0.635 ± 0.052 | 0.621 ± 0.047 |
| Florida | 0.685 ± 0.038 | 0.690 ± 0.031 |
| Payoya | 0.701 ± 0.035 | 0.676 ± 0.033 |
| Negra Serrana | 0.599 ± 0.043 | 0.625 ± 0.040 |
| Retinta | 0.631 ± 0.058 | 0.624 ± 0.053 |
| **Northern Spain** | | |
| Azpi Gorri | 0.651 ± 0.037 | 0.653 ± 0.036 |
| Blanca de Rasquera | 0.607 ± 0.050 | 0.641 ± 0.047 |
| **Balearic Islands** | | |
| Eivissenca | 0.613 ± 0.041 | 0.638 ± 0.036 |
| Mallorquina | 0.628 ± 0.036 | 0.684 ± 0.030 |
| **Canary Islands** | | |
| Ajuy | 0.637 ± 0.036 | 0.644 ± 0.024 |
| Majorera | 0.659 ± 0.033 | 0.665 ± 0.024 |
| Palmera | 0.482 ± 0.039 | 0.494 ± 0.038 |
| Tinerfeña del Norte | 0.582 ± 0.050 | 0.572 ± 0.035 |
| Tinerfeña del Sur | 0.624 ± 0.039 | 0.623 ± 0.034 |
| **Cape Verde** | | |
| Cape Verde | 0.630 ± 0.041 | 0.680 ± 0.028 |
| **Northwest Africa** | | |
| Morocco | 0.702 ± 0.035 | 0.699 ± 0.033 |
| Tunisia | 0.654 ± 0.031 | 0.703 ± 0.031 |
| Algeria | 0.692 ± 0.039 | 0.683 ± 0.034 |
| **Egypt** | | |
| Barki | 0.676 ± 0.049 | 0.656 ± 0.040 |
| Balaidi | 0.678 ± 0.042 | 0.706 ± 0.035 |
| Saidi | 0.710 ± 0.033 | 0.718 ± 0.027 |
| Anglo-Nubian | 0.610 ± 0.029 | 0.635 ± 0.028 |
| **Nigeria** | | |
| Maradi | 0.671 ± 0.037 | 0.682 ± 0.036 |
| West African Dwarf | 0.638 ± 0.044 | 0.668 ± 0.040 |
| Sahelian | 0.655 ± 0.045 | 0.674 ± 0.040 |
| **Southern Africa** | | |
| Boer | 0.602 ± 0.030 | 0.600 ± 0.023 |
| Kalahari | 0.601 ± 0.043 | 0.643 ± 0.030 |

Supplementary Table S2. Genetic distance matrix of pairwise F_ST_ values relative to goat populations from ten geographic areas

| **Population** | **Southern**  **Spain** | **Northern**  **Spain** | **Balearic**  **Islands** | **Canary**  **Islands** | **Cape Verde** | **Nigeria** | **C. West**  **Europe** | **South**  **Africa** | **Northwest**  **Africa** |
| --- | --- | --- | --- | --- | --- | --- | --- | --- | --- |
| **Northern Spain** | 0.023 | - |  |  |  |  |  |  |  |
| **Balearic Islands** | 0.016 | 0.035 | - |  |  |  |  |  |  |
| **Canary Islands** | 0.086 | 0.1 | 0.102 | - |  |  |  |  |  |
| **Cape Verde** | 0.061 | 0.079 | 0.087 | 0.064 | - |  |  |  |  |
| **Nigeria** | 0.050 | 0.068 | 0.057 | 0.066 | 0.070 | - |  |  |  |
| **C. West Europe** | 0.033 | 0.044 | 0.049 | 0.109 | 0.065 | 0.073 | - |  |  |
| **South Africa** | 0.128 | 0.130 | 0.137 | 0.143 | 0.127 | 0.110 | 0.139 | - |  |
| **Northwest Africa** | 0.038 | 0.048 | 0.049 | 0.077 | 0.049 | 0.031 | 0.061 | 0.096 | - |
| **Egypt** | 0.055 | 0.062 | 0.068 | 0.091 | 0.059 | 0.058 | 0.065 | 0.103 | 0.036 |

Supplementary Table S3. Coalescent estimates of mutation-scaled population sizes (θ = 4N_e_μ) and migration rates (M_ij_ = m_ij_/μ) obtained with Migrate-n (the averaged results of two independent runs are presented) and their 95% confidence limits.

Abbreviations: NSPAIN: Northern Spanish goats, CW EUROPE: Central-Western European goats, SSPAIN: Southern Spanish goats, NW AFRICA: Northwestern African goats EGYPT: Egyptian goats, CANARY I: Canarian goats.

| **Population 1** | **Population 2** | **θ_1_ and 95% CI** | | **θ_2_ and 95% CI** | | **M_2>1_ and 95% CI** | | **M_1>2_ and 95% CI** | |
| --- | --- | --- | --- | --- | --- | --- | --- | --- | --- |
| NSPAIN | CW EUROPE | 0.96 | 0.82 - 1.14 | 0.77 | 0.58 - 0.97 | 9.19 | 6.65 – 11.63 | 16.47 | 12.29 - 21.06 |
| SSPAIN | NW AFRICA | 0.75 | 0.63 - 0.87 | 0.90 | 0.78 - 1.03 | 19.50 | 17.64 - 20.92 | 15.13 | 13.08 - 16.86 |
| NW AFRICA | EGYPT | 0.89 | 0.71-1.11 | 0.95 | 0.84-1.10 | 20.74 | 18.74-23.32 | 17.39 | 15.11-20.18 |
| CANARY I. | NW AFRICA | 0.61 | 0.52 - 0.71 | 0.97 | 0.79 - 1.18 | 10.29 | 8.32 -12.73 | 4.42 | 2.88 - 5.93 |

Supplementary Table S4. Coalescent estimates of mutation-scaled population sizes (θ = 4N_e_μ) and migration rates (M_ij_ = m_ij_/μ) obtained with IMa and their 95% confidence limits.

Abbreviations: NSPAIN: Northern Spanish goats, CW EUROPE: Central-Western European goats, SSPAIN: Southern Spanish goats, NW AFRICA: Northwestern African goats EGYPT: Egyptian goats, CANARY I: Canarian goats.

| **Population 1** | **Population 2** | **θ_1_ and 95% CI** | | **θ_2_ and 95% CI** | | **M_2>1_ and 95% CI** | | **M_1>2_ and 95% CI** | |
| --- | --- | --- | --- | --- | --- | --- | --- | --- | --- |
| NSPAIN | CW EUROPE | 0.85 | 0 - 3.30 | 4.43 | 0 - 7.43 | 4.16 | 0 - 28.20 | 0.0002 | 0 - 7.24 |
| SSPAIN | NW AFRICA | 0.54 | 0 - 2.62 | 4.83 | 0 - 8.68 | 8.93 | 1.50 - 41.04 | 0.06 | 0 – 5.00 |
| NW AFRICA | EGYPT | 5.00 | 0 - 8.93 | 4.02 | 0 - 8.51 | 0.015 | 0 - 3.18 | 3.52 | 0 - 8.59 |
| CANARY I. | NW AFRICA | 0.59 | 0 - 1.41 | 4.95 | 0 - 8.40 | 6.32 | 1.99 - 20.45 | 0.092 | 0 - 2.37 |

Supplementary Table S5. IMa estimates and 95% confidence intervals (CI) of times of divergence (expressed as a median) and ancestral population sizes (q_A_)

In this table, time of divergence is expressed as the product of splitting time (in years) by the mutation rate per year.. The ancestral population size q_A_ should be understood as four times the product of the effective size of the ancestral population by the mutation rate. Abbreviations: NSPAIN: Northern Spanish goats, CW EUROPE: Central-Western European goats, SSPAIN: Southern Spanish goats, NW AFRICA: Northwestern African goats EGYPT: Egyptian goats, CANARY I: Canarian goats.

| **Population 1** | **Population 2** | **q_A_ and 95% CI** | | **t and 95% CI** | |
| --- | --- | --- | --- | --- | --- |
| NSPAIN | CW EUROPE | 12.45 | 0.012 – 100 | 0.51 | 0.33 -18.75 |
| SSPAIN | NW AFRICA | 23.10 | 0 - 60.77 | 0.68 | 0.17 - 4.05 |
| NW AFRICA | EGYPT | 26.22 | 9.67 – 145.40 | 1.55 | 0.39 – 13.94 |
| CANARY I. | NW AFRICA | 16.94 | 6.49 -53.40 | 0.35 | 0.17 - 13.80 |

Supplementary Table S6. Goat populations sampled in the current study

| **Population** | **Breed** | **Acronym** | **Sample size** |
| --- | --- | --- | --- |
| Central West Europe | Saanen | SAAN | 36 |
|  | Alpine | ALPI | 37 |
|  | **TOTAL** | - | **73** |
| Southern Spain | Blanca Andaluza | BLAN | 40 |
|  | Blanca Celtibérica | CELT | 40 |
|  | Malagueña | MALA | 40 |
|  | Murciano-Granadina | MURC | 40 |
|  | Florida | FLOR | 50 |
|  | Payoya | PAYO | 36 |
|  | Negra Serrana | SERR | 42 |
|  | Retinta | RETI | 15 |
|  | **TOTAL** | - | **303** |
| Northern Spain | Blanca de Rasquera | RASQ | 50 |
|  | Azpi Gorri | AZPI | 56 |
|  | **TOTAL** | - | **106** |
| Balearic Islands | Eivissenca | EIVI | 79 |
|  | Mallorquina | MALL | 58 |
|  | **TOTAL** | - | **137** |
| Canary Islands | Ajuy | AJUY | 55 |
|  | Majorera | MAJO | 85 |
|  | Palmera | PALM | 47 |
|  | Tinerfeña del Norte | TINN | 53 |
|  | Tinerfeña del Sur | TINS | 70 |
|  | **TOTAL** | - | **310** |
| Cape Verde | Cape Verde | CVER | **37** |
| Northwest Africa | Morocco | MORO | 26 |
|  | Tunisia | TUNI | 59 |
|  | Algeria | ALGE | 11 |
|  | **TOTAL** |  | **96** |
| Egypt | Barki | BARK | 44 |
|  | Baladi | BALA | 31 |
|  | Saidi | SAID | 40 |
|  | Anglo-Nubian | NUBI | 41 |
|  | **TOTAL** | - | **156** |
| Nigeria | Maradi | MARA | 47 |
|  | West African Dwarf | WEAF | 67 |
|  | Sahelienne | SAHE | 47 |
|  | **TOTAL** | - | **161** |
| Southern Africa | Boer | BOER | 46 |
|  | Kalahari Red | KALA | 47 |
|  | **TOTAL** | - | **93** |
| **Total number of individuals** | | | **1,472** |

Supplementary Table S7. List of microsatellite markers employed to analyse the genetic variability of Spanish and African goat breeds

| Marker | Primer Sequences (5' - 3') | Fluorescence |
| --- | --- | --- |
| *BM1329* | TTG TTT AGG CAA GTC CAA AGT C  AAC ACC GCA GCT TCA TCC | NED |
| *BM1818* | AGC TGG GAA TAT AAC CAA AGG  AGT GCT TTC AAG GTC CAT GC | FAM |
| *BM6506* | GCA CGT GGT AAA GAG ATG GC  AGC AAC TTG AGC ATG GCA C | HEX |
| *BM6526* | CAT GCC AAA CAA TAT CCA GC  TGA AGG TAG AGA GCA AGC AGC | FAM |
| *BM8125* | CTC TAT CTG TGG AAA AGG TGG G  GGG GGT TAG ACT TCA ACA TAC G | FAM |
| *CSRD247* | GGA CTT GCC AGA ACT CTG CAA T  CAC TGT GGT TTG TAT TAG TCA GG | FAM |
| *CSRM60* | AAG ATG TGA TCC AAG AGA GAG GCA  AGG ACC AGA TCG TGA AAG GCA TAG | NED |
| *CSSM66* | ACA CAA ATC CTT TCT GCC AGC TGA  AAT TTA ATG CAC TGA GGA GCT TGG | HEX |
| *ETH10* | GTT CAG GAC TGG CCC TGC TAA CA  CCT CCA GCC CAC TTT CTC TTC TC | FAM |
| *ETH225* | GAT CAC CTT GCC ACT ATT TCC T  ACA TGA CAG CCA GCT GCT ACT | NED |
| *HAUT27* | TTT TAT GTT CAT TTT TTG ACT GG  AAC TGC TGA AAT CTC CAT CTT A | NED |
| *HSC* | CTG CCA ATG CAG AGA CAC AAG A  GTC TGT CTC CTG TCT TGT CAT C | HEX |
| *ILSTS11* | GCT TGC TAC ATG GAA AGT GC  CTA AAT TGC AGA GCC CTA CC | HEX |
| *ILSTS19* | AGG GAC CTC ATG TAG AAG C  ACT TTT GGA CCC TGT AGT GC | HEX |
| *INRA005* | CAA TCT GCA TGA AGT ATA AAT AT  CTT CAG GCA TAC CCT ACA CC | HEX |
| *INRA063* | ATT TGC ACA AGC TAA ATC TAA CC  AAA CCA CAG AAA TGC TTG GAA G | HEX |
| *MAF065* | AAA GGC CAG AGT ATG CAA TTA GGA G  CCA CTC CTC CTG AGA ATA TAA CAT G | NED |
| *MAF209* | GAT CAC AAA AAG TTG GAT ACA ACC GTG G  TCA TGC ACT TAA GTA TGT AGG ATG CTG | HEX |
| *McM527* | GTC CAT TGC CTC AAA TCA ATT C  AAA CCA CTT GAC TAC TCC CCA A | NED |
| *MM12* | CAA GAC AGG TGT TTC AAT CT  ATC GAC TCT GGG GAT GAT GT | NED |
| *OarFCB011* | GGC CTG AAC TCA CAA GTT GAT ATA TCT ATC AC  GCA AGC AGG TTC TTT ACC ACT AGC ACC | FAM |
| *OarFCB048* | GAG TTA GTA CAA GGA TGA CAA GAG GCA C  GAC TCT AGA GGA TCG CAA AGA ACC AG | FAM |
| *OarFCB304* | CCC TAG GAG CTT TCA ATA AAG AAT CGG  CGC TGC TGT CAA CTG GGT CAG GG | HEX |
| *SPS115* | AAA GTG ACA CAA CAG CTT CTC CAG  AAC GAG TGT CCT AGT TTG GCT GTG | NED |
| *SRCRSP05* | GGA CTC TAC CAA CTG AGC TAC AAG  TGA AAT GAA GCT AAA GCA ATG C | HEX |
| *SRCRSP08* | TGC GGT CTG GTT CTG ATT TCA C  CCT GCA TGA GAA AGT CGA TGC TTA G | NED |
| *SRCRSP23* | TGA ACG GGT AAA GAT GTG  TGT TTT TAA TGG CTG AGT AG | FAM |
| *SRCRSP24* | AGC AAG AAG TGT CCA CTG ACA G  TCT AGG TCC ATC TGT GTT ATT GC | HEX |
| *TGLA122* | CCC TCC TCC AGG TAA ATC AGC  AAT CAC ATG GCA AAT AAG TAC ATA C | FAM |

Fig. S1. Structure analysis (K = 2-6) of 32 goat populations from Spain and Africa.

**Southern Spain** (red): Blanca Andaluza (BLAN), Blanca Celtibérica (CELT), Malagueña (MALA), Murciano-Granadina (MURC), Florida (FLOR), Payoya (PAYO), Serrana (SERR), Retinta (RETI).

**Northern Spain**: Azpi-Gorri (AZPI), Blanca de Rasquera (RASQ).

**Balearic Islands**: Eivissenca (EIVI), Mallorquina (MALL).

**Canary Islands**: Ajuy (AJUY), Majorera (MAJO), Palmera (PALM), Tinerfeña del Norte (TINN), Tinerfeña del Sur (TINS).

**Cape Verde** (CVER).

**Central West Europe**: Saanen (SAAN), Alpine (ALPI).

**Northwest Africa**: Moroccan (MORO), TUNE (Tunisian), Algerian (ALGE).

**Egypt**: Barki (BARK), Baladi (BALA), Saidi (SAID);

**Nigeria**: Maradi (MARA), West African Dwarf (WEAF), Sahel (SAHE).

**South Africa**: Boer (BOER), Kalahari Red (KALA).

The Anglo-Nubian breed (mixed British, African and Asian origins) is indicated as NUBI.


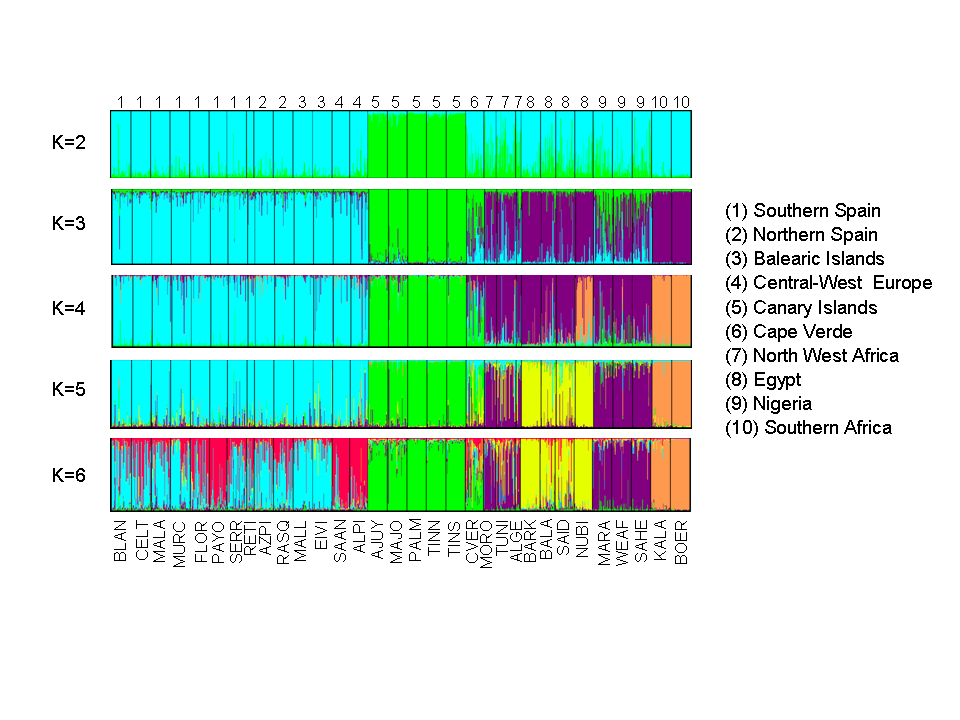


Fig. S2. The method of Evanno (Mol Ecol. 14:2611-20, 2005) indicated that the most likely number of clusters was K = 4, followed by K = 3.


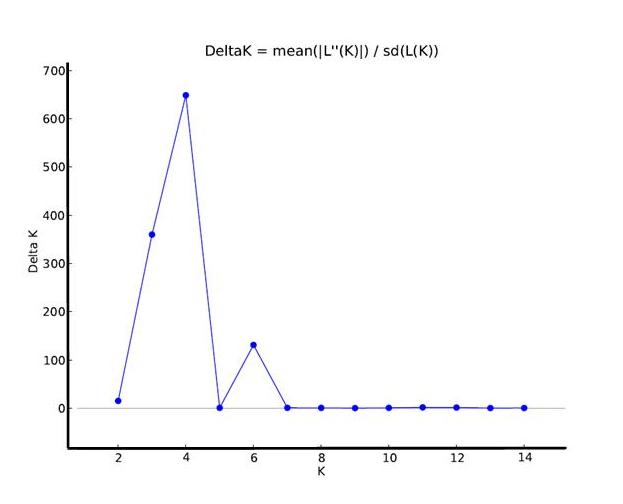


Fig. S3. Coefficients of membership (K = 4) of African and Spanish goat populations. Anglo-Nubian goats were not included in the Egyptian group because they have a complex Indian, African and British ancestry. Europe: Central West Europe (Saanen and Alpine).


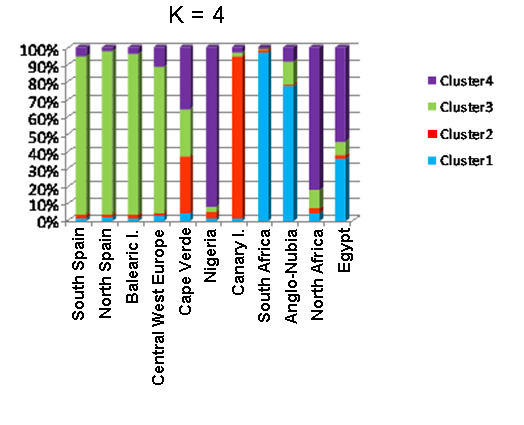


Fig. S4a. Average estimated sample sizes and autocorrelations of IMa runs (M-mode) and posterior density plots of theta (q_1_ and q_2_) and migration (m1 = M21 and m2 = M12) parameters.


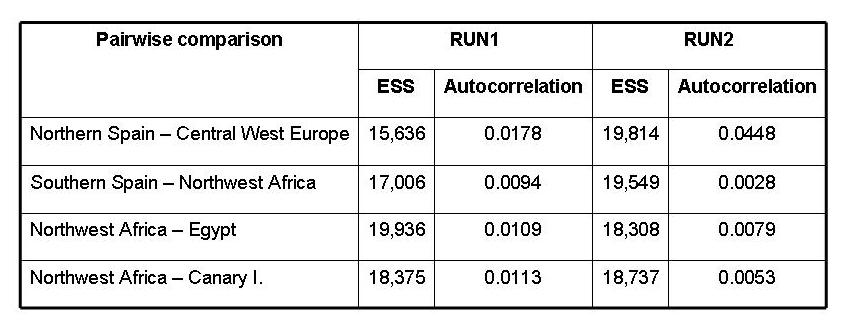


Fig. S4b. Posterior density plots of theta (q1 and q2) and migration (m1 = M21 and m2 = M12) parameters (Northern Spain and Central West European goat populations). Parameter estimates obtained in two independent M-mode runs are shown. Graphs picturing approximate posterior densities correspond to the L-mode analysis. Prior ranges based on preliminar runs: q_1_= 5, q_2_ = 10, m_1_ = 50, m_2_ = 20.


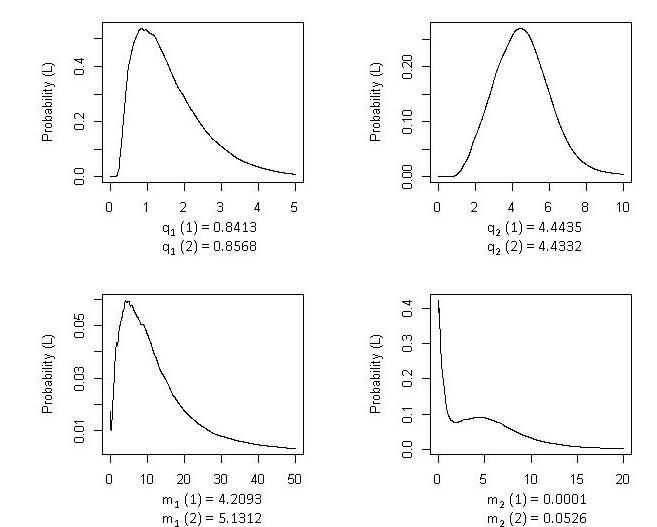


Fig. S4c. Posterior density plots of theta (q1 and q2) and migration (m1 = M21 and m2 = M12) parameters (Southern Spain and Northwest African goat populations). Parameter estimates obtained in two independent M-mode runs are shown. Graphs picturing approximate posterior densities correspond to the L-mode analysis. Prior ranges based on preliminar runs: q_1_= 5, q_2_ = 20, m_1_ = 100, m_2_ = 5.

.
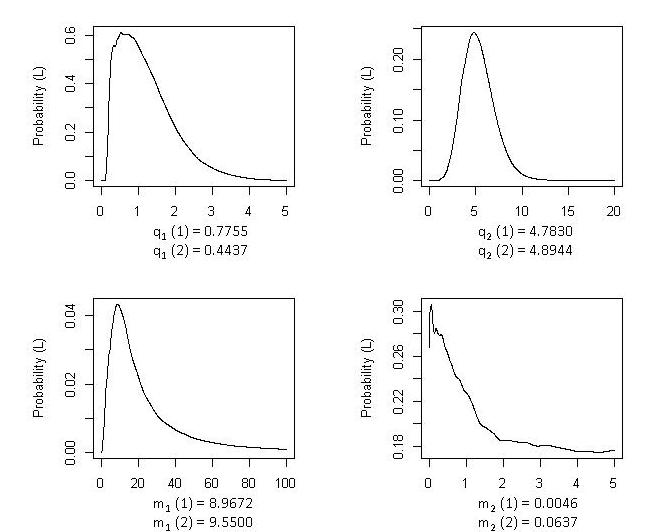


Fig. S4d. Posterior density plots of theta (q1 and q2) and migration (m1 = M21 and m2 = M12) parameters (Northwest African and Egyptian goat populations). Parameter estimates obtained in two independent M-mode runs are shown. Graphs picturing approximate posterior densities correspond to the L-mode analysis. Prior ranges based on preliminar runs: q_1_= 15, q_2_ = 15, m_1_ = 15, m_2_ = 15.


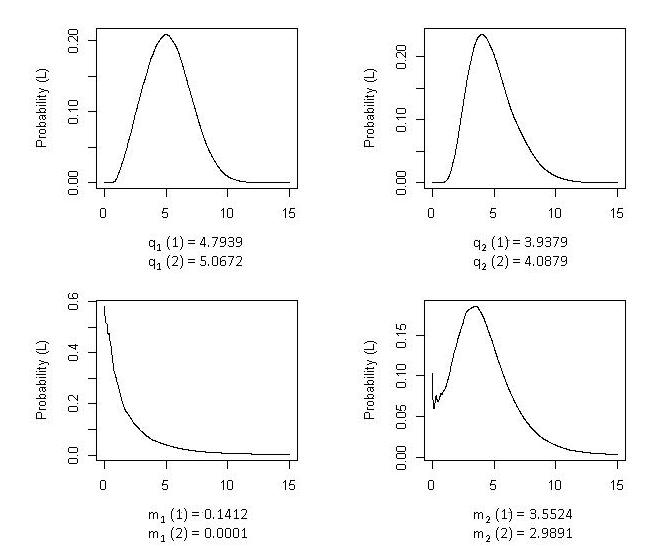


Fig. S4e. Posterior density plots of theta (q1 and q2) and migration (m1 = M21 and m2 = M12) parameters (Northwest African and Canarian goat populations). Parameter estimates obtained in two independent M-mode runs are shown. Graphs picturing approximate posterior densities correspond to the L-mode analysis. Prior ranges based on preliminar runs: q_1_= 5, q_2_ = 20, m_1_ = 50, m_2_ = 5.


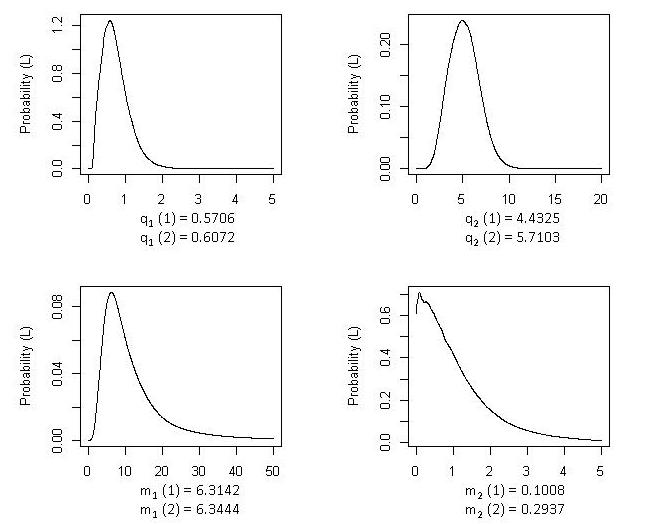


Supplementary Fig. S5a. Geographic distribution of the populations sampled in Spain. Saanen and Alpine goats were sampled in Southern Spain, but these two breeds have French and Swiss origins, respectively (we have classified them as Central West European).

The map of Spain, https://commons.wikimedia.org/wiki/File: Blank Spain Map (Provinces).svg is licensed under the Creative Commons Attribution-Share Alike 3.0 Unported license. The license terms can be found on the following link: https://creative commons.org/licenses/by-sa/3.0/deed.en.


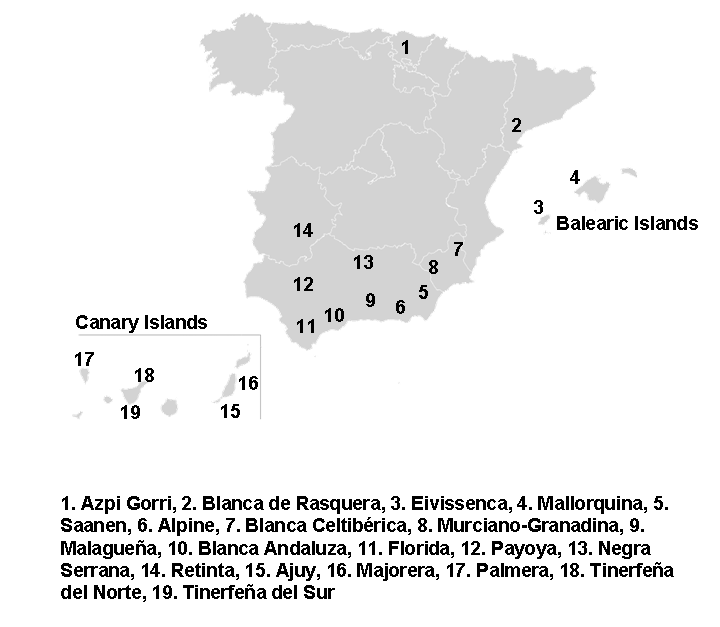


Supplementary Fig. S5b. Geographic distribution of the populations sampled in Africa.

The map of Africa, https://commons. wikimedia.org /wiki/File: File:Blank Map-Africa.svg, is licensed under the Creative Commons Attribution-Share Alike 3.0 Unported license. The license terms can be found on the following link: https://creative commons.org/licenses/by-sa/3.0/deed.en.


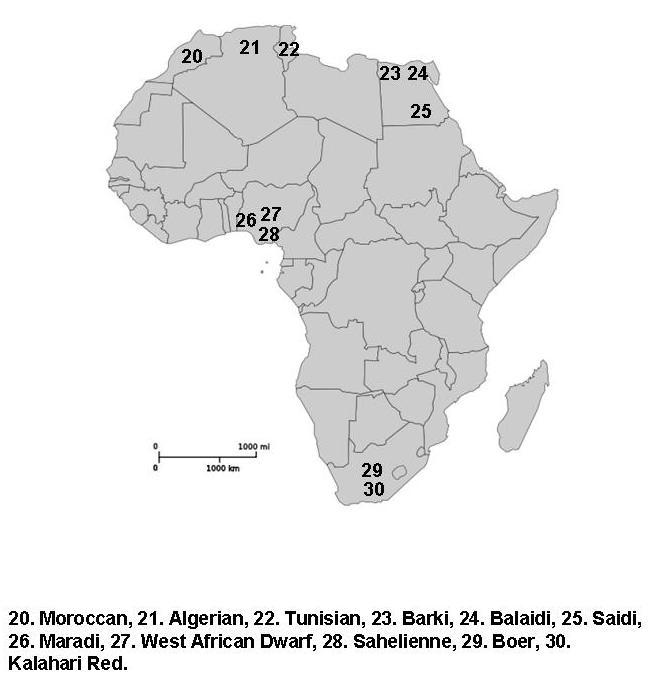

Supplement: Supplementary Information [file srep38935-s1.doc]
